# Exploring Cancer Metastasis: From Mechanisms to Treatments and Beyond

**DOI:** 10.1002/cnr2.70201

**Published:** 2025-04-24

**Authors:** Mukul S. Godbole, Amruta Naik

**Affiliations:** ^1^ Department of Biosciences and Technology School of Science and Environmental Studies, Dr. Vishwanath Karad MIT World Peace University Pune India

**Keywords:** cancer, cancer progression, cancer therapeutics, mechanism of action, metastasis, microbiomes

Metastasis is an important attribute of cancer cells. If and when the primary tumor microenvironment becomes less conducive for growth and sustenance, metastatic growth allows cancer cells to explore potentially more conducive environments for survival and territorial expansion, instead of perishing at the original site. Cancer cells utilize multiple cellular mechanisms for processes such as detaching from the initial tumor site (migration and invasion), intravasation (crossing the endothelial barrier), circulation (movement through blood and/or lymph), extravasation (exit from circulation into a secondary tissue), and colonization (establishment of micro‐metastases). These processes are further supported by angiogenesis (development of new blood vessels), immune evasion (overcoming immune regulation), modulation of primary and secondary tissue microenvironments (modulation of tumor‐infiltrating immune cell activity, suppression of antitumor effects, etc.), secretion of tissue growth factors supporting metastases, and evasion of cell death. Moreover, the smooth transitions and interlinks between each of these processes adds to the complexity of metastasis and, hence, makes it difficult and crucial to target the processes that allow systemic spread of cancer cells. The “Cancer Metastasis: Mechanisms and Treatment” special issue primarily focuses on discussing the various mechanisms that drive metastasis of cancer cells to regional or distant organs and explores the strategies to target these metastatic processes, ultimately aiming to improve patient outcomes. We believe that the research and review articles published as part of the special issue would collectively aid in improving our current understanding and allow more critical research in the field. Here, we provide glimpses of all articles published in the special issue and encourage the readers to further dwell into the intricacies discussed in each of the articles.

Breast cancer, known for its high prevalence and tumor heterogeneity among women, frequently metastasizes to distant organs, such as the brain, lungs, liver, lymph nodes, and bones, leading to poor survival outcomes. Unfortunately, traditional treatments such as chemotherapy, radiotherapy, endocrine therapy, and immunotherapy show limited success in patients with metastatic breast cancer. A review article by Naik and Godbole offers a comprehensive discussion on the interesting, yet underexplored, roles of gut and breast microbiomes in influencing breast cancer metastasis, particularly to the bone [1]. The article elaborates on the mechanisms by which microorganisms either promote or abrogate breast cancer metastasis, such as epithelial–mesenchymal transition, immune modulation in the bone microenvironment, enhanced cancer cell survival in circulation due to bacteria‐induced altered cytoskeletal architecture, intratumoral persistence of bacteria in metastasizing cancer cells, altered steroid hormone metabolism, and abrogated tumor microenvironment. Further, the review discusses the potential feasibility of applying adjuvant treatment strategies targeting microbiomes that have been tested in other cancers, including probiotics and prebiotics, species‐specific antibiotics, antibiotic‐based elimination of intracellular bacteria, plant‐based natural compounds, and fecal microbiota transplantation to curb metastasis of breast cancer to the bone. The article also highlights the importance of maintaining a diverse gut microbiome to prevent dysbiosis, which may otherwise trigger breast cancer and metastasis. Taken together, the review encourages further research and discussions on targeting the gut and breast microbiomes to overcome breast cancer growth and metastasis, especially to the bone.

Triple‐negative breast cancer (TNBC), an aggressive subtype of breast cancer, has an enhanced propensity for distant metastasis that contributes to poor prognosis and frequent relapse in patients. While chemo–radiotherapy can help contain the growth of the primary tumor, resistance to these treatments and tumor recurrence—due to several genetic and epigenetic factors—are commonly observed in patients with TNBC. One key factor contributing to the resistance is an increased expression of epidermal growth factor receptor (EGFR). Kar et al. addressed this issue by identifying small molecule inhibitors targeting EGFR, which, as shown in the study, is highly expressed in breast cancer stem cells (BCSCs) [2]. The authors employed molecular docking to identify potential EGFR inhibitors and rigorously evaluated their cytotoxic, anti‐migratory, and pro‐apoptotic potentials in vitro. Specifically, compound 1e significantly enhanced the inhibitory effects of doxorubicin on TNBC cell proliferation, migration, tumorigenesis, and apoptosis in vitro, and tumor growth in an orthotopic mouse model. Thus, the study suggests that targeting EGFR in BCSCs could improve the efficacy of existing chemotherapeutic compounds and potentially overcome drug resistance in aggressive, metastatic breast cancer.

Small molecule inhibitors, a category of targeted therapy, are designed to specifically and selectively bind proteins and inhibit their functions. Despite the development of approximately 100 small molecule inhibitors reported so far, the complete plethora of proteins, especially kinases and transcription factors, is yet to be targeted in cancer. A study by Koll et al. explained the therapeutic potential of small molecule 4 (Sm4) that targets the transcription factor SOX18 in lymphangiosarcoma and cancer‐induced lymphangiogenesis [3]. Cancer cells often release growth factors that enhance lymphangiogenesis, in both primary tumors and sentinel lymph nodes, and thus facilitate lymph node metastasis. Lymphangiosarcoma, a lymphatic cancer, is characterized by poor survival outcomes and limited treatment options, highlighting the need for more effective therapies. The in vitro study demonstrated that treatment with Sm4 significantly reduced SOX18 transcript and protein levels in human dermal lymphatic endothelial cells and lymphangiosarcoma cells. Sm4 also suppressed key lymphatic phenotype markers and inhibited cell migration, without affecting cell viability. These promising results suggest that inhibiting SOX18 with Sm4 may serve as a novel therapeutic approach for the treatment of lymphangiosarcoma and cancer‐induced lymphatic metastasis.

Statins, a class of drugs that inhibit the activity of hydroxymethylglutaryl‐CoA reductase (HMG‐CoA), have traditionally been used for the treatment of some medical conditions due to their capacity to reduce levels of cholesterol, triglycerides, and low‐density lipoprotein. However, recent studies have suggested their potential application as anticancer agents, particularly due to the reliance of cancer cells on the cholesterol metabolic pathway, irrespective of their statuses. A review article by Tripathi et al. discusses the multifaceted applications of statins, with a special emphasis on their anticancer effects [4]. Statins, by effectively inhibiting HMG‐CoA reductase in the mevalonate pathway, compromise the functionality of cancer cell membranes and thus impede their growth and metastatic potential. The review highlights that cancer cells that are reliant on the mevalonate pathway for growth may particularly be responsive to inhibition by statins. Statins tend to induce apoptosis via the BCL2 signaling pathway, regulate the cell cycle via the p53–YAP axis, and modulate epigenetic changes, such as altering CpG island methylation and histone acetylation. The authors explain that these mechanisms underline the potential chemo‐preventive effects of statins, especially by reducing tumor relapse and improving survival outcomes in patients undergoing long‐term therapy with statins. However, despite the encouraging descriptions, the authors caution the need for extensive population‐based clinical studies with larger patient cohorts and longer follow‐up durations to definitively establish statins as anticancer agents. The review builds a compelling narrative for repurposing statins as cholesterol‐lowering and potential anticancer agents to enhance the efficacy of conventional therapies.

Immune cells in the microenvironment of epithelial cancers play a key role in shaping the nature of cancer and their responses to anticancer treatment, especially as the presence of immunosuppressive cells changes the tumor dynamics. The review article by Tamuli et al. offers a comprehensive exploration of the intricate dynamics of the tumor microenvironment of solid tumors and their pivotal role in modulating immune cell functions [5]. The review elucidates the mechanisms by which non‐immunosuppressive macrophages and gamma delta (γδ) T cells are reprogrammed into potent immunosuppressors within the tumor milieu, while enhancing the immune‐suppressive capacities of myeloid‐derived suppressor cells (MDSCs) after their infiltration into the tumor microenvironment. Further, the authors explain that tumor‐associated macrophages, monocytic MDSCs (M‐MDSCs) and γδ T cells frequently develop robust immunosuppressive traits in epithelial malignancies and thus significantly hinder the natural antitumor immune responses mounted by tumor‐infiltrating T and B lymphocytes. This immunosuppressive transformation of M‐MDSCs and γδ T cells is linked with poor prognoses of patients with various epithelial cancers. The review provides valuable insights into the mechanisms by which such immune cells gain immunosuppressive abilities and contribute to the development of cancer metastasis. Finally, the review proposes potential therapeutic interventions that may aid in effectively ensuring adaptive immune responses in the fight against epithelial cancers.

Small cell lung cancer (SCLC) is one of the aggressive malignancies with neuroendocrine nature and highly metastatic potential. SCLC tumors present with a high propensity for therapy resistance due to insufficiently understood metastasis mechanisms to distant organs such as the bones, brain, liver, and lymph nodes. A review article by He [6] describes the various mechanisms by which SCLC metastasizes to distant organs. Specifically, the review highlights the involvement of the tumor immune microenvironment in developing a prometastatic niche; VEGF‐induced vasculogenesis, along with vascular mimicry; epithelial–mesenchymal transition involving repression of E‐cadherin and silencing of Notch; migration of SCLC cells due to increased expression and activity of nuclear factor I/B, B1 integrin, and selectin; and factors such as PLGF and CXCR4 that allow SCLC to form metastases specifically in the bones and brain, respectively. The review also discusses the potential of miR‐1 as a therapeutic target for metastatic SCLC. However, the narrative underscores the need for deeper exploration of metastasis‐associated targets. Taken together, a comprehensive understanding of the mechanisms is crucial for developing effective interventions to curb SCLC progression and improve patient outcomes.

We believe that appreciating the facts and thinking beyond conventions is the first step towards overcoming research hurdles. The articles featured in the ‘Cancer Metastasis: Mechanisms and Treatment’ special issue present significant strides in cancer research, discuss the shortcomings/limitations of current strategies and areas worthy of investigation, and offer newer insights into potential therapeutic opportunities to prevent cancer metastasis. From elucidating the roles of microbiomes and tumor‐infiltrating immune cells to repurposing existing drugs and developing novel inhibitors to combat metastasis, these studies collectively contribute to a better understanding of the evolving landscape of treatments targeting cancer metastasis. As we continue to unravel the complexities of cancer biology, such research endeavors bring us a step closer to achieving more effective cancer therapies.

## Author Contributions

**Mukul S. Godbole:** writing – original draft, writing – review and editing. **Amruta Naik:** writing – original draft, writing – review and editing.

## Conflicts of Interest

The authors declare no conflicts of interest.

## Data Availability

Data sharing is not applicable to this article as no new data were created or analyzed in this study.

## References

[1] A. Naik and M. S. Godbole , “Elucidating the Intricate Roles of Gut and Breast Microbiomes in Breast Cancer Metastasis to the Bone,” Cancer Reports 7, no. 8 (2024): e70005, 10.1002/cnr2.70005. 39188104 PMC11347752

[2] T. Kar , P. Dugam , S. Shivhare , et al., “Epidermal Growth Factor Receptor Inhibition Potentiates Chemotherapeutics‐Mediated Sensitization of Metastatic Breast Cancer Stem Cells,” Cancer Reports 7, no. 3 (2024): e2049, 10.1002/cnr2.2049. 38522013 PMC10961089

[3] K. K. Koll , M. M. Zimmermann , P. A. Will , U. Kneser , and C. Hirche , “The Transcription Factor SOX18 Inhibitor Small Molecule 4 Is a Potential Treatment of Cancer‐Induced Lymphatic Metastasis and Lymphangiosarcoma,” Cancer Reports 8, no. 1 (2025): e70110, 10.1002/cnr2.70110. 39791369 PMC11726641

[4] S. Tripathi , E. Gupta , and S. Galande , “Statins as Anti‐Tumor Agents: A Paradigm for Repurposed Drugs,” Cancer Reports 7, no. 5 (2024): e2078, 10.1002/cnr2.2078. 38711272 PMC11074523

[5] B. Tamuli , S. Sharma , M. Patkar , and S. Biswas , “Key Players of Immunosuppression in Epithelial Malignancies: Tumor‐Infiltrating Myeloid Cells and γδ T Cells,” Cancer Reports 7, no. 5 (2024): e2066, 10.1002/cnr2.2066. 38703051 PMC11069128

[6] C. He , “Activating Invasion and Metastasis in Small Cell Lung Cancer: Role of the Tumour Immune Microenvironment and Mechanisms of Vasculogenesis, Epithelial‐Mesenchymal Transition, Cell Migration, and Organ Tropism,” Cancer Reports 7, no. 10 (2024): e70018, 10.1002/cnr2.70018. 39376011 PMC11458887

